# Long-term follow-up of prophylactic mesh reinforcement after emergency laparotomy. A retrospective controlled study

**DOI:** 10.1186/s12893-021-01243-x

**Published:** 2021-05-18

**Authors:** A. Bravo-Salva, N. Argudo-Aguirre, A. M. González-Castillo, E. Membrilla-Fernandez, J. J. Sancho-Insenser, L. Grande-Posa, M. Pera-Román, J. A. Pereira-Rodríguez

**Affiliations:** 1 Servicio de Cirugía General Y del Aparato Digestivo, Parc de Salut Mar, Hospital del Mar, P. Marítim 23-25, 08003 Barcelona, Spain; 2 Departament de Ciències, Experimentals I de La Salut, Universitat Pompeu Fabra, Dr. Aiguader 88, 08003 Barcelona, Spain; 3 Departament de Cirurgia, Vall d’Hebrón, Unitat Departamental Parc de Salut Mar, Universitat Autónoma de Barcelona, Passeig Vall d’Hebrón 119-129, 08035 Barcelona, Spain; 4 Departament de Ciències Morfològiques, Universitat Autónoma de Barcelona, Campus Bellaterra, 08193 Cerdanyola del Vallès - Barcelona, Spain

**Keywords:** Hernia prevention, Emergency Surgery, Prophylactic mesh, Contaminated surgery and long-term follow up

## Abstract

**Background:**

Prevention of incisional hernias with a prophylactic mesh in emergency surgery is controversial. The present study aimed to analyze the long-term results of prophylactic mesh used for preventing incisional hernia after emergency midline laparotomies.

**Methods:**

This study was a registered (NCT04578561) retrospective analysis of patients who underwent an emergency midline laparotomy between January 2009 and July 2010 with a follow-up period of longer than 2 years. Long-term outcomes and risk factors for the development of incisional hernias between patients who received a prophylactic reinforcement mesh (Group M) and suture (Group S) were compared.

**Results:**

From an initial 266 emergency midline laparotomies, 187 patients were included. The median follow-up time was 64.4 months (SD 35). Both groups had similar characteristics, except for a higher rate of previous operations (62 *vs.* 43.2%; *P* = 0.01) and operation due to a revision laparotomy (32.5 vs.13%; *P* = 0.02) in the M group. During follow-up, 29.9% of patients developed an incisional hernia (Group S 36.6% vs. Group M 14.3%; *P* = 0.002). Chronic mesh infections were diagnosed in 2 patients, but no mesh explants were needed, and no patient in the M group developed chronic pain. Long-term risk factors for incisional hernia were as follows: smoking (HR = 2.47; 95% CI 1.318–4.624; *P* = 0.05), contaminated surgery (HR = 2.98; 95% CI 1.142–7.8; P = 0.02), surgical site infection (SSI; HR = 3.83; 95% CI 1.86–7.86; *P* = 0.001), and no use of prophylactic mesh (HR = 5.09; 95% CI 2.1–12.2; *P* = 0.001).

**Conclusion:**

Incidence of incisional hernias after emergency midline laparotomies is high and increases with time. High-risk patients, contaminated surgery, and surgical site infection (SSI) benefit from mesh reinforcement. Prophylactic mesh use is safe and feasible in emergencies with a low long-term complication rate.

*Trial registration:* NCT04578561. www.clinicaltrials.gov

## Background

In the era of minimally invasive surgery, midline laparotomies are still a common approach in emergencies [1]. This type of incision leads to a high incidence of incisional hernias (IHs) when compared with other approaches such as lateral laparotomies [2–4]. When a midline laparotomy is performed in high-risk patients, this incidence increases; one of these high-risk situations is emergency surgery, and the incidence of IHs after an emergency midline laparotomy (EML) ranges from 18 to 54% [5–7]. It has been demonstrated that prophylactic mesh reinforcement after midline elective laparotomy is useful in reducing IH, especially in high-risk patients [8–13].

Emergency surgery is a well-known risk factor for IHs, but its prevention has been poorly studied, and the main point investigated thus far has been the closure technique [14]. In a recent study, a standardized technique of closure showed a reduction in the incidence of IHs and burst abdomen [15, 16]. Mesh reinforcement in emergencies is controversial, and there is a lack of high-quality studies on this issue. On the one hand, a recent meta-analysis concluded that there was inadequate evidence to recommend its use in a standardized approach [17]; on the other hand, a randomized control trial demonstrated that prophylactic mesh in EMLs reduced the incidence of dehiscence fascia when analyzing short-term outcomes, but with a higher incidence of surgical site infection (SSI) in the mesh group [18]. Surgical site occurrences (SSO) and acute and chronic infection are the main concerns for surgeons when implementing a prophylactic mesh, especially in the emergency setting, apart from other complications such as chronic pain and intestinal fistulas [19]. Despite these concerns, there have been no studies evaluating the long-term complications of mesh reinforcement in EMLs, although some recent long-term studies in elective cases have demonstrated its safety and efficacy in reducing the incidence of IHs with a long-term follow up [20]. The European Hernia Society Guidelines (EHS) recommendation is to use prophylactic mesh in high-risk patients, but no recommendation is given for the closure of EMLs due to a lack of data [21]. Hence, we hypothesize that prophylactic mesh may also be useful in EMLs to prevent IHs.

The main objective of this study was to analyze long-term results and complications after prophylactic mesh implantation during emergency midline laparotomies.

## Methods

We designed a retrospective analysis following the STROBE guideline statements [22] for a cohort of patients who underwent emergency midline laparotomies in the period between January 2009 and July 2010 in a University Hospital. We included all consecutive patients from January 2009 to July 2010 with at least 2 years of follow-up and compared those who received a prophylactic onlay mesh (Group M) with those in whom the laparotomy was closed only with suture (Group S).

The eligibility criteria were as follows: undergoing emergency midline laparotomy excluding those with concomitant hernia repair, those who received incisions outside the midline, those with delayed abdominal closure, those who died in the immediate postoperative period, and a minimum 2 years of follow-up.

Surgical technique: Closure of incision was performed by running suture of double loop number 1 polydioxanone (PDS, Ethicon, Bridgewater, NJ), and in some cases, retention polypropylene number 1 (Ethilon, Ethicon) suture was used. In group M, prophylactic synthetic partially absorbable lightweight large pore mesh (Ultrapro, Ethicon) placed ‘‘onlay’’ was used as reinforcement [23]. The overlap involved 3-cm wide on each side of the incision and superior and inferior incisional limits. Mesh fixation was performed using a double crown of fascial staples (DFS; Autosuture, Covidien, MA), with additional polypropylene 2/0 stitches (Prolene; Ethicon) In all mesh group patients, closed suction onlay drains were placed.

All postoperative evaluations were performed by clinical appointment with abdominal exploration from a surgeon. The definition of an IH was according to the description of the EHS: “Any abdominal wall gap with or without a bulge in the area of a postoperative scar perceptible or palpable by clinical examination or imaging” [24]. IH diagnosis was determined through clinical exploration, and in cases of diagnostic uncertainty, abdominal ultrasound (US) or computed tomography (CT) was performed. In addition, all the abdominal CTs or USs performed during the follow-up due to other causes (oncological follow-up, for instance) were revised for asymptomatic IH detection [25, 26]. Chronic pain was defined as significant pain persisting after 3 months of intervention [27, 28]. Chronic mesh infection was defined as the presence of local symptoms such as inflamed skin, chronic leaking, fistula, abscess, or prosthetic exposure, persisting despite conservative medical management including antibiotics and local care [29].

In the statistical analysis, numeric variables were presented as mean and standard deviation, and categorical variables were reported as proportions. The association between qualitative variables was assessed using contingency tables (chi-squared test and Fisher’s exact tests, when necessary), and the quantitative tests were conducted using Student’s t-test for unpaired data or the Mann–Whitney U test when necessary. The normality of the distribution of numeric variables was assessed using normal QQ plots. Odds ratios (OR) and confidence intervals (CI) for IH incidence in univariate analysis were calculated for each group.

A multivariate analysis of the risk factors for IHs in the general cohort was conducted. The predictive capacity of each variable and its independence were analyzed using a Cox regression model. Survival curves for hernia-free survival were estimated by a Cox survival analysis method in the whole cohort. Statistical analysis was performed using Software package SPSS v.20.0 (IBM Inc, Rochester, MN).

The Local Clinical Research Ethics Committee approval was obtained and the observational study protocol was registered with the NCT04578561 code of ClinicalTrials.gov database [30].

## Results

**Fig. 1:**
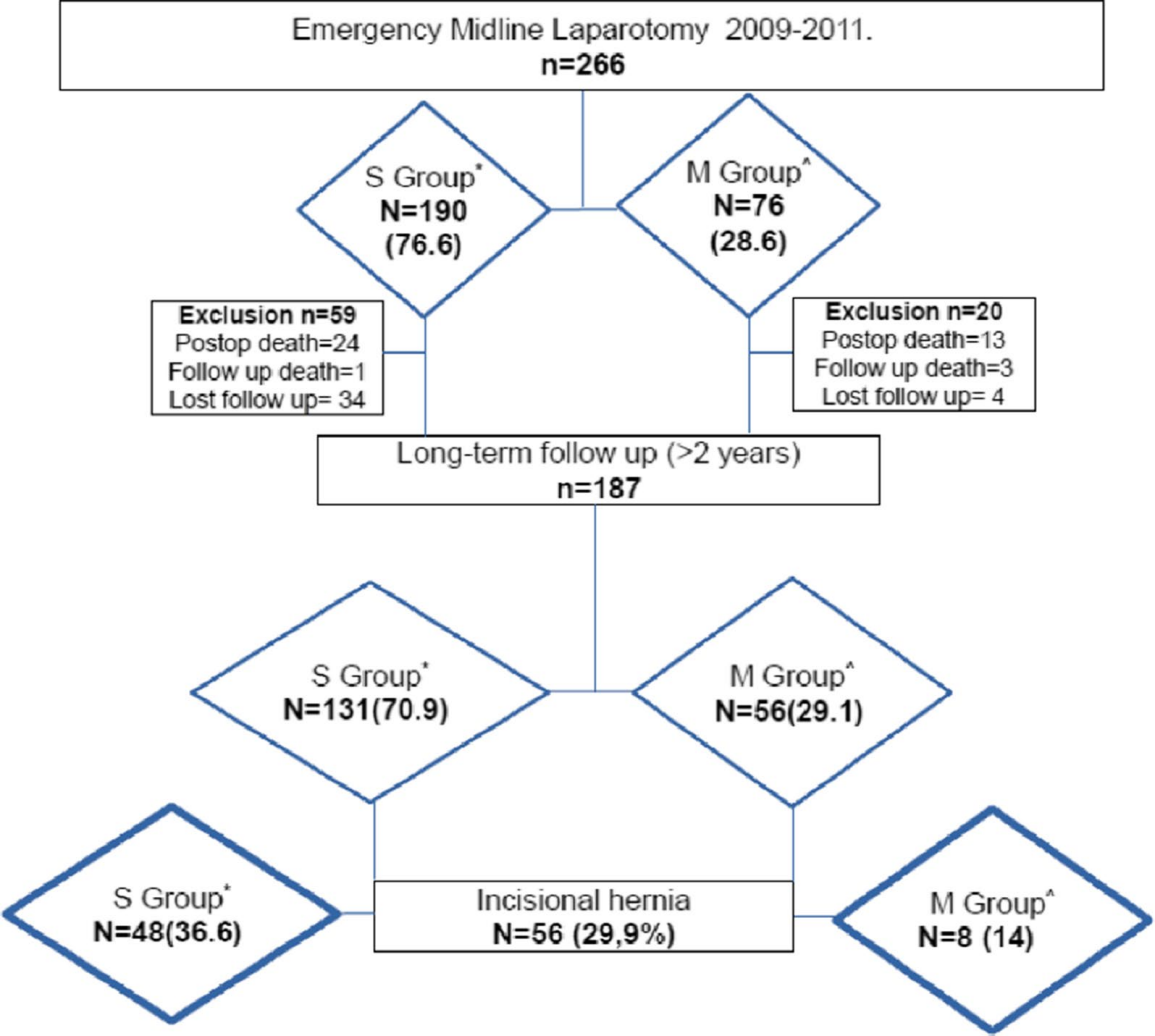
Study’s flow chart

A total of 187 patients from the initial cohort of 266 patients were included. The causes for exclusion were as follows: 37 postoperative death, 4 death during follow-up (non-mesh related), and 38 lost to follow-up (Fig. 1). The median follow-up period was 64.4 months (SD 35.2). The demographic characteristics of the patients, operations, and diagnostics are shown in Table 1. Both groups showed similar characteristics, except for higher rate of previous abdominal operations (62 vs. 43.2%; *P* = 0.02) and revision laparotomy as indication (32.5 vs.15%; *P* = 0.02) in group M.

**Table 1:** Comparative analysis of demographics, comorbidities, and surgery characteristics

	Total (N = 187)	Group M (N = 56)	Group S (N= 131)	*P* value
Demographic variables				
Age,yr (SD)	65.4 (16.8)	68.39(15.2)	64.16(17.34)	0.51
Sex male/female (%)	84/103 (44.9/55.1)	23/33 (27.4/32)	61/70(72.6/68)	0.29
ASA score I-II/III-IV	82/105 (43.9/56.1)	21/35 (25.6/33.3)	61/70(74.4/66.7)	0.71
Risk factors for incisional hernia (%)				
Smoking	71 (30)	20 (35.2%)	51 (38.9)	0.67
COPD	29 (15.3)	12 (21.4)	17 (13)	0.11
DM	33 (17.6)	24 (18.3)	9 (16.1)	0.71
Inmunosupresion	20 (10.7)	14 (10.7)	6 (10.7)	0.99
Previous abdominal operation	92 (49.2)	35 (62)	57 (43.2)	0.01
Obesity ( BMI > 30)	62 (34.4)	21 (38.9)	41 (32.5)	0.41
Operative Diagnostics (%)				
Neoplasm	38 (20.3)	11 (29.6)	27 (20.6)	0.57
Obstruction	42 (22.5)	19 (45.2)	23 (54.3)	0.14
Peritonitis	72 (38.5)	21 (37.5)	51 (38.8)	0.85
Hemorrhage	23 (12.3)	4 (7.1)	19 (14.5)	0.16
Ischemia	11 (5.9)	1 (1.8)	10 (7.6)	0.12
Type of surgery				
Revision laparotomy (%)	49 (26.2)	21 (32.5)	28 (15)	0.02

Long-term results of the follow-up are summarized in Table 2. A total of 56 (29.9%) IHs were diagnosed, of which 41 were diagnosed during short-term and 15 during long-term follow-up (8 in the M group vs. 48 in the S group; *P* = 0.002). From 38 patients where additional retention sutures were used (37 in S group and 1 in M group) 15 IHs were diagnosed in those patients (39.5% of the subgroup patients) all of them from S group. In long-term follow up 78(41.1%) patients had radiological exploration.

**Table 2:** Long-Term Outcomes (> 2 year follow-up)

N (%)	General; N = 187 (100)	Group M; N = 56 (29.1)	Group S; N = 131 (70.9)	*P* value
Incisional hernia	56 (29.9)	8(14.3)	48 (36.6)	0.002
Long-term incisional hernia diagnosis*	15 (32.1)	3 (37.5)	12 (28.2)	0.53
Chronic pain	0	0	–	–
Chronic infection	2 (1.3)	2 (4.3)	–	–
Mesh removal	0	0	–	–
Incisional hernia repair	14 (25.0)	2 (25.0)	12 (25.0)	0.03
Recurrence after incisional hernia repair	3 (21.4)	2 (100)	1 (8.3)	0.39

*IH diagnosis after 2 years of postoperative follow-up

During follow-up, 14 IHs (25%) were repaired, 12 in the S group and two in the M group; three recurrences occurred: two in the M group and one in the S group. The causes of avoiding/delaying hernia repair were as follows: 10 patients had no clinical symptoms and IH was diagnosed in revision CTs, 9 patients died during follow-up for non-mesh-related complications without hernia repair, 8 patients received watchful waiting management due to an absence of symptoms and/or severe comorbidities, seven patients rejected hernia repair, six patients were lost to follow-up after IH diagnosis, and two IHs appeared after a new iterative laparotomy during the follow-up period.

Chronic mesh infections were diagnosed in two patients; both patients were treated without the need for surgical mesh removal, and complete healing was achieved at 4 and 12 months after surgery. In both cases, mesh removal was offered but rejected by the patients due to the high risk of surgical complications. One case of chronic wound infection was a patient with colon resection due to obstructive sigmoid tumor, and in the second case, the patient received small bowel resection and adhesiolysis. Both patients had high intraoperative peritoneal contamination, and both presented postoperative SSI that was treated initially with debridement and saline irrigations. Both patients achieved healing at 4th and 12th postoperative months. None of the patients of both groups were diagnosed with chronic pain during follow-up.

The incidence and risk of IH by subgroup analysis is shown in Table 3. Wide differences in the incidence were observed in the analysis of high-risk subgroups. In operations related to cancer, no incidence of hernia was diagnosed in the M group as compared to 44.4% incidence (*P* = 0.008) in the S group. In patients with SSI, only one patient in the M group had IH (7.7%) as compared to 17 patients (73.9%; *P* < 0.001) in the S group. In the other subgroups, the use of prophylactic mesh had a statistically significant lower incidence of IH, except in obese patients where no statistical differences (*p* = 0.054) were observed, but the incidence was also lower.

**Table 3:** Incidence and risk (not using prophylactic mesh) of incisional hernia by long-term follow-up of subgroups

	General; N = 187	Group M; N = 56 (29.1)	Group S; N = 131 (70.9)	OR	95% CI	*P* value
General (187)	56 (29.9)	8 (14.3)	48 (36.6)	2.56	1.3–5.06	0.002
Contaminated Surgery (groups III-IV*; N = 138)	48 (34.8)	6 (15)	42 (42.9)	2.85	1.32–6.18	0.002
Peritonitis (N = 72)	27 (37.5)	23 (45.1)	4 (19)	2.36	0.93–6.01	0.038
Cancer (N = 38)	12 (31.6)	0	12 (44.4)	–	–	0.008
SSI (N = 36)	18 (50)	1 (7.7)	17 (73.9)	9.63	1.43–64.15	< 0.001
Revision laparotomy (N = 49)	18 (36.7)	3 (14.3)	15 (53.7)	3.75	1.24–11.29	0.005
Obesity (N = 62)	22 (35.5)	4 (19)	18 (53.3)	2.31	0.894–5.94	0.053
Age; > 70 yr. (N = 85)	20 (23.5)	3 (10)	17 (30.9)	3.09	0.985–9.7	0.03

*World Health Organization wound contamination classification [40], *yr* years

Multivariate analysis of the risk factors for IHs were as follows: smoking (HR = 2.47; 95% CI 1.318–4.624; *P* = 0.05), contaminated surgery (groups III–IV*) (HR = 2.985 h; 95% CI 1.142–7.8; *P* = 0.02), SSI (HR = 3.829; 95% CI 1.86–7.86; *P* = 0.001), and no use of prophylactic mesh (HR = 5.093 h; 95%CI 2.13–12.17; *P* = 0.001). Table 4.

**Table 4:** Incisional hernia risk factors in long-term follow up. Multivariate Cox regression

	B	SE	Wald (Chi-square)	HR	95,0% CI		*P* value
					Inferior	Superior	
Patient related							
Smoking	0.904	0.320	7.959	2.468	1.318	4.624	0.05
Surgery related							
No prophylactic mesh	1.628	0.444	13.421	5.093	2.132	12.167	0.001
Contaminated Surgery (groups III-IV*)	1.094	0.490	4.981	2.985	1.142	7.8	0.02
Complication related							
Surgical site infection	1.343	0.367	13.375	3.829	1.865	7.864	0.001

*III-IV contaminated stage (contaminated or dirty) according to World Health Organization wound contamination classification [40]

**Fig. 2:**
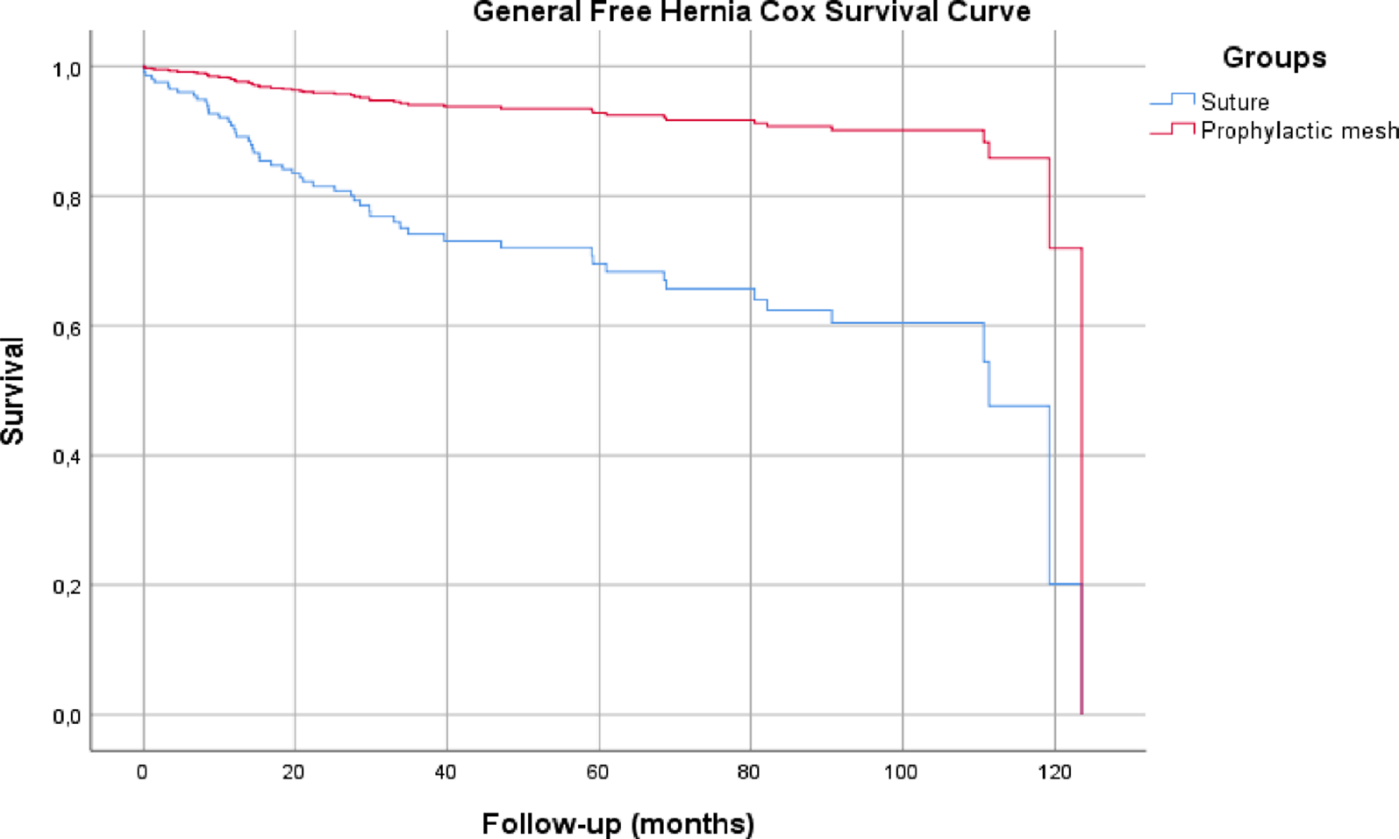
General free hernia COX survival curve

Hernia-free survival curves showed a clearly higher incidence of IHs in the S group. Median hernia-free survival was 37 ± 35.96 months in the S group *vs.* 54 ± 38 months in the M group; *P* = 0.001 (Fig. 2).

## Discussion

Emergency laparotomy is frequently associated with worst outcomes in severely ill patients and with a higher risk of burst abdomen and IH. As such, prophylactic measures for these patients are more relevant to prevent these complications.

Few data are available on long-term outcomes after the use of prophylactic mesh in an emergency setting. To our knowledge, the present study is the first study to analyze the long-term results of IH prevention with mesh in emergency midline laparotomies. One of the strengths of our study is that the median follow-up time was longer than 5 years in 70% of the cohort.

The characteristics of the patients in both groups showed small differences. There were more patients in the M group with a revision laparotomy or with previous midline incision, both of which are well-known risk factors for burst abdomen [12] and IH, and probably, this circumstance is the explanation for the higher frequency of using a mesh for prevention. This measure was clearly effective in revision laparotomy as there was a reduction in the incidence of IHs (14.3% M group vs 53.7% S group; CI: 1.24–11.29; O.R 3.75; *P* = 0.005).

In our results, almost one third of the diagnosed IHs appeared after 2 years of follow-up. Therefore, studies with a shorter term would probably underestimate the real incidence of IHs. Our data support similar results obtained in other studies over a long-term follow-up period for elective patients [20].

Despite the use of mesh reinforcement to prevent IH, it seems that IH is delayed but does not disappear completely as observed in hernia-free survival analysis by the parallelism of accumulated incidence lines. Hence, the use of a mesh as a prophylaxis seems to act as a “palliative” more than a curative measure; therefore, we believe that new closure, prevention techniques, and mesh materials should be investigated to evaluate their long-term outcomes.

When an IH appears despite previous use of a prophylactic mesh and needs to be repaired, the risk of recurrence seems to be high, in our study where all incisional hernia repair in M group patient had a recurrence. The presence of a previous mesh could be related to a more difficult operation, but the small size of the sample precludes to make conclusions.

In the present study, the number of patients analyzed was higher than that in our previous analysis [11]. This is because in the previous study, some patients had not reached the minimum follow-up for the study inclusion (1 year), whereas in the present study, those patients were included as they exceeded the follow-up time.

The use of synthetic mesh in contaminated environments, as in emergency laparotomies, is controversial [17], but other studies have demonstrated its safety in contaminated complex ventral hernia repairs [31]. Our results support the view that concerns regarding mesh infections are exaggerated, and the risk of mesh-related complications is minimal. This also confirms our previous results on the safety and efficacy of the use of synthetic mesh in emergency midline laparotomy reinforcement, even in the presence of peritonitis [11], thus ensuring that mesh reinforcement maintains its capability to prevent IHs with a minimum rate of complications after a long-term follow-up.

Prophylactic mesh significantly reduces IHs, even when used in high-risk subgroups [32]. All the analyzed subgroups (Table 3) seemed to benefit, and only in obese patients, the incidence of IHs was higher (53.3%) in the S group but without reaching statistical significance (OR 2.31, 0.894–5.94, *P* = 0.053) probably due to the sample size.

The use of retention sutures had no influence on reducing IHs, and moreover, had a higher incidence of IHs when used (39.5%). These results are similar to those of other studies that evaluated retention sutures [33] and strongly supports their discontinuation as a closure method.

In our study, the closure of aponeurosis was not performed using the “small bites” technique as the benefits of this technique to reduce IH [34] have not yet been published, and moreover, this study was conducted in patients with elective operations. However, some evidence has been recently published, and the “small bites” technique also seems to be useful in emergency laparotomy [15, 16]; however, more investigation on this topic is needed.

One of the main warnings against the use of prophylactic mesh in emergencies is the risk of SSI and chronic mesh infection as emergency surgical fields are commonly associated with contamination. Fear of mesh colonization with its complications have pushed some groups to search for alternatives such as biological or absorbable meshes in contaminated ventral hernia repairs [35–38]. In our study, there was low incidence of such complications, and the capability of mesh prevention was specifically useful in contaminated surgeries and in the presence of postoperative SSI. Both were independent risk factors for IH after emergency midline laparotomies closed with a suture, with a high power of influence (HR 2.98 and 3.82, respectively). Hence, we believe that the use of prophylactic synthetic onlay mesh reinforcement in high-risk patients, including those with contaminated surgeries, after the closure of an emergency midline laparotomy is a good prevention measure due to a simple technique, although it is mandatory to conduct further high-quality studies as prospective or randomized control trials to confirm this. Type of fixation had no differences in IH incidence, however other types of fixation such as fibrin sealants or biologic glues could be interesting to analyze, as had been useful in other studies. [39].

The use of a mesh in high-risk patients in elective midline laparotomies has a strong recommendation from the EHS guidelines [21] and is a cost-effective measure [40]. The use of onlay position is effective in preventing IHs and is easy and rapid to perform for a General Surgeon when compared with other mesh insertion planes, such as the retrorectus plane [23], which is known to have a lower complication rate in prophylactic abdominal wall reinforcements [41].

## Conclusions

In conclusion, the rate of IHs after emergency midline laparotomies is high and increases with time, even when using a prophylactic mesh. High-risk patients, contaminated surgeries, and SSIs clearly benefit from mesh reinforcement with a low IH rate and long-term complications. Prophylactic mesh in the emergency setting to prevent IHs seems to be a safe and feasible procedure, supported by long-term evaluation.

## Data Availability

The datasets generated and analyzed during the current study are available from the corresponding author on reasonable request.

## Abbreviations

BMI: Body mass index
CI: Confident interval
CT: Computed tomography
HR: Hazard ratio
IHs: Incisional hernias
EHS: European Hernia Society.
EML: Emergency midline laparotomy
SD: Standard deviation
SSI: Surgical site infection
SSO: Surgical site occurrences
OR: Odds ratio
US: Ultrasound sonography
Yr: Year
